# The optimized carbapenem inactivation method for objective and accurate detection of carbapenemase-producing *Acinetobacter baumannii*

**DOI:** 10.3389/fmicb.2023.1185450

**Published:** 2023-07-13

**Authors:** Sirui Zhang, Peng Mi, Jingdan Wang, Pu Li, Kai Luo, Shuyan Liu, Mona Mohamed Al-Shamiri, Jin’e Lei, Simin Lai, Bei Han, Yanjiong Chen, Lei Han, Shaoshan Han

**Affiliations:** ^1^Department of Microbiology and Immunology, School of Basic Medical Sciences, Xi’an Jiaotong University Health Science Center, Xi’an, China; ^2^Department of Laboratory Medicine, Shaanxi Provincial People’s Hospital, Xi’an, China; ^3^School of Public Health, Xi’an Jiaotong University Health Science Center, Xi’an, China; ^4^Department of Laboratory Medicine, The First Affiliated Hospital of Xi’an Jiaotong University, Xi’an, China; ^5^Department of Hepatobiliary Surgery, The First Affiliated Hospital of Xi’an Jiaotong University, Xi'an, China

**Keywords:** *Acinetobacter baumannii*, carbapenemase, modified carbapenem inactivation method, optimized carbapenem inactivation method, minimal inhibitory concentrations, carbapenemases gene expression

## Abstract

The modified carbapenem inactivation method (mCIM) recommended by the Clinical and Laboratory Standards Institute is not applicable for detecting carbapenemases in *Acinetobacter baumannii*. Four currently reported phenotypic detection methods, namely, the modified Hodge test, the mCIM, the adjusted mCIM, and the simplified carbapenem inactivation method (sCIM), did not perform well in our 90 clinical *A. baumannii* isolates. Thus, the minimal inhibitory concentrations (MICs) of carbapenems and the existence and expression of carbapenemase-encoding genes were detected to explain the results. According to the E-test, which was more accurate than the VITEK 2 system, 80.0 and 41.1% were resistant to imipenem (IPM) and meropenem (MEM), respectively, and 14.4 and 53.3% exhibited intermediate resistance, respectively. Five β-lactamase genes were found, of which *bla*_OXA-51-like_, *bla*_TEM_, and *bla*_OXA-23-like_ were detected more frequently in 85 non-susceptible strains. The expression of *bla*_OXA-23-like_ was positively correlated with the MIC values of IPM and MEM. Therefore, an improved approach based on the mCIM, designated the optimized CIM (oCIM), was developed in this study to detect carbapenemases more accurately and reproducibly. The condition was improved by evaluating the factors of *A. baumannii* inoculum, incubation broth volume, and MEM disk incubation time. Obvious high sensitivity (92.94%) and specificity (100.00%) were obtained using the oCIM, which was cost-effective and reproducible in routine laboratory work.

## 1. Introduction

*Acinetobacter baumannii* is one of the most important opportunistic pathogens, and so is one of the main causes of hospital-acquired infections (Dijkshoorn et al., 2007). This species has a strong ability to acquire antimicrobial resistance, thus producing various types of resistant isolates, including multidrug-resistant (MDR), extensively drug-resistant (XDR), and even pandrug-resistant (PDR) strains. Carbapenems were formerly the last-resort antibiotics for the treatment of MDR *A. baumannii* infections. However, an increasing number of carbapenem-resistant *A. baumannii* (CRAB) strains have been found throughout the world, posing one of the greatest known threats to modern medicine (Hamidian and Nigro, 2019). Although different kinds of resistance mechanisms have been discovered in CRAB, the acquisition of carbapenemases results in the most striking activity against carbapenems (Da Silva and Domingues, 2016).

The accurate and rapid detection of carbapenemases is crucial for clinical diagnosis. Although several molecular methods, such as specific PCR, commercial microarrays, whole genome sequencing, and matrix-assisted laser desorption ionization time-of-flight mass spectrometry (MALDI-TOF MS), can be used to identify carbapenemases (Nordmann and Poirel, 2019), a reliable phenotypic method would be indispensable in clinics due to its advantages, which include the fact that phenotypic methods are not limited by the type of carbapenemases, have a lower cost, and are convenient for hospitals without the machines required for the molecular methods listed above.

A number of phenotypic methods for screening carbapenemase activity in Gram-negative bacteria have been reported (van der Zwaluw et al., 2015; Pierce et al., 2017), whereas there are no standard methods recommended for *A. baumannii* by the Clinical and Laboratory Standards Institute (CLSI). The most frequently used methods published by the CLSI to determine carbapenemase production are the modified Hodge test, the Carba NP test, and the modified carbapenem inactivation method (mCIM). However, the modified Hodge test is only recommended for *Enterobacterales* (CLSI, 2016) and is even excluded from the 28th edition of “Performance standards for antimicrobial susceptibility testing” (CLSI, 2018). The mCIM can be applied for *Enterobacterales* and *Pseudomonas aeruginosa*, but not for *A. baumannii* (CLSI, 2017, 2018, 2022). Moreover, although the Carba NP test was performed for *Acinetobacter* spp. in previous years, this method is no longer recommended (CLSI, 2018). Therefore, an appropriate method is necessary for detecting carbapenemases in *A. baumannii*.

Several modified methods have been reported to identify carbapenemase-producing, including CIMTris (Uechi et al., 2017), adjusted mCIM (Vu et al., 2020), the CarbAcineto NP test (Dortet et al., 2014), and the simplified carbapenem inactivation method (sCIM; Jing et al., 2018). However, none of them are officially recommended by CLSI. This may be due to the shortcomings of these tests, such as false positives (CIMTris) and less sensitivity or specificity (mCIM, Carba NP, CarbAcineto NP, and sCIM) when tested in clinical *A. baumannii* isolates (Simner et al., 2018; Vu et al., 2020; Khuntayaporn et al., 2021). In addition, our research group obtained less reliable results in *A. baumannii* strains using some of the mentioned above methods, which were mainly based on mCIM. As a result, this study aimed to develop a further optimized carbapenem inactivation method (oCIM) to provide a more accurate method for the diagnosis of carbapenemase-producing *A. baumannii*.

## 2. Materials and methods

### 2.1. Bacterial strains and cultural conditions

Ninety *A. baumannii* clinical isolates were collected from hospitalized patients in Shaanxi Provincial People’s Hospital in Xi’an during 2018. Isolates were confirmed as *A. baumannii* using MALDI-TOF MS. The quality control strain *Escherichia coli* ATCC 25922, the carbapenemase-positive (CP) control strain *Klebsiella pneumoniae* strain ATCC BAA-1705, and the carbapenemase-negative (CN) control strain *Klebsiella pneumoniae* ATCC BAA-1706 were stored in the laboratory. All strains were grown on Luria-Bertani (LB, Beijing Land Bridge, Beijing, China) agar plates and preserved in LB broth containing 20% glycerol at −80°C for further analysis. Muller–Hinton agar (MHA, OXOID, Hampshire, United Kingdom) and trypticase soy broth (TSB, Beijing Land Bridge) were used in this study as required for the experiments. Ten micrograms of meropenem (MEM) and imipenem (IPM) disks (BIO-KONT, Wenzhou, China) were used for carbapenemase detection.

### 2.2. Antimicrobial susceptibility testing

Antimicrobial susceptibility testing was performed using the VITEK 2 system (bioMérieux, Marcyl’étoile, France). The minimal inhibitory concentrations (MICs) of IPM and MEM were verified using E-test strips (BIO-KONT) three times, with the antibiotic concentrations ranging from 0.008 to 32 μg/mL. The susceptibility results were interpreted according to the M100-S28 guidelines published by the CLSI as follows: ≥ 8 μg/mL was resistant, 4 μg/mL was intermediate, and ≤ 2 μg/mL was sensitive (CLSI, 2022).

### 2.3. Phenotypic tests for carbapenemase detection by four reported methods

Four different phenotypic methods, among which the majority were based on the mCIM, were performed to determine the production of carbapenemases in clinical isolates, including the modified Hodge test (CLSI, 2016) and mCIM (CLSI, 2018) described by the CLSI, the adjusted mCIM reported by Vu et al. (2020), and the sCIM reported by Jing et al. (2018). Negative controls were treated following the same procedures but without bacterial isolates. Experiments were performed in triplicate. The effectiveness of these methods for the confirmation of carbapenemase activity was compared and evaluated. The evaluation criteria for the modified Hodge test results referred to the CLSI (2017). For the mCIM and adjusted mCIM, positive results were regarded as results with an inhibition zone diameter at 6–15 or 16–18 mm with colonies. The negative results were considered results with a zone diameter ≥ 19 mm. The indeterminate results were interpreted as having zone diameter within 16–18 mm or zone diameter ≥ 19 mm with colonies in the zone (CLSI, 2018; Khuntayaporn et al., 2021). For the sCIM, an inhibition zone diameter of 6–20 or ≤ 22 mm with satellite colonies was considered to be a positive result. A zone diameter ≥ 26 mm was regarded as a negative result, while a zone diameter of 23–25 mm was interpreted as an indeterminate result (Jing et al., 2018).

### 2.4. Investigation of β-lactamase-encoding genes

The genomic DNA of all clinical isolates was extracted using the TIANamp Bacteria DNA Kit (TIANGEN, Beijing, China) according to the manufacturer’s instructions. Based on relevant research reports, the existence of 14 β-lactamases genes was detected via PCR, including five class A genes (*bla*_TEM_, *bla*_SHV_, *bla*_CTX_, *bla*_KPC_, and *bla*_GES_), four class B genes (*bla*_IMP_, *bla*_VIM_, *bla*_SIM-1_, and *bla*_NDM_), and five class D genes (*bla*_OXA-23-like_, *bla*_OXA-24-like_, *bla*_OXA-48-like_, *bla*_OXA-51-like_, and *bla*_OXA-58-like_). Primers are listed in Table 1. PCR was performed using 2 × Taq Master Mix (Novoprotein, Suzhou, China) in a total volume of 20 μL, containing 10 μL of 2 × Taq Master Mix, 0.5 μM of each primer, and 2 μL of DNA template. The amplification program consisted of predenaturation at 94°C for 2 min, followed by 30 cycles of denaturation at 95°C for 20 s, annealing at the appropriate temperature for 20 s (see Table 1), extension at 72°C for 1 min, and a final elongation at 72°C for 5 min. The PCR results are further utilized as the gold standard to evaluate the sensitivity of different phenotypic methods (Abouelfetouh et al., 2019).

**Table 1:** Primers used in this study.

Primer	Primer sequence (5′–3′)		Annealing temperature	Product size (bp)	Reference
*bla*_KPC_	F	TGTCACTGTATCGCCGTC	56°C	1,010	Khan et al. (2017)
	R	CTCAGTGCTCTACAGAAAACC			
*bla*_GES_	F	GTTTTTGCAATGTGCTCAACG	54°C	371	Nie (2013)
	R	TGCCATAGCAATAGGCGTAG			
*bla*_TEM_	F	ATAAAATTCTTGAAGACGAAA	55°C	1,079	Nie (2013)
	R	GACAGTTAGCAATGCTTAATCA			
*bla*_SHV_	F	GCCTTTATCGGCCCTCACTCAAG	56°C	897	This study
	R	TTAGCGTTGCCAGTGCTCGATCA			
*bla*_CTX_	F	CGTCACGCTGTTGTTAGGAA	56°C	823	Nie (2013)
	R	ACCGTCGGTGACGATTTTAG			
*bla*_IMP_	F	GAAGGCGTTTATGTTCATAC	52°C	587	Jean et al. (2015)
	R	GTACGTTTCAAGAGTGATGC			
*bla*_VIM_	F	GTTTGGTCGCATATCGCAAC	52°C	389	Jean et al. (2015)
	R	AATGCGCAGCACCAGGATAG			
*bla*_SIM-1_	F	TACAAGGGATTCGGCATCG	54°C	571	Nie (2013)
	R	TAATGGCCTGTTCCCATGTG			
*bla*_NDM_	F	GCAGCTTGTCGGCCATGCGGGC	52°C	782	Jean et al. (2015)
	R	GGTCGCGAAGCTGAGCACCGCAT			
*bla*_OXA-23-like_	F	GATGTGTCATAGTATTCGTCG	52°C	1,067	Nie (2013)
	R	TCACAACAACTAAAAGCACTG			
*bla*_OXA-24-like_	F	GGTTAGTTGGCCCCCTTAAA	52°C	246	Nie (2013)
	R	AGTTGAGCGAAAGGGGATT			
*bla*_OXA-48-like_	F	GCGTGGTTAAGGATGAACAC	52°C	438	Jean et al. (2015)
	R	CATCAAGTTCAACCCAACCG			
*bla*_OXA-51-like_	F	ATGAACATTAAAGCACTC	52°C	825	Nie (2013)
	R	CTATAAAATACCTAATTGTTC			
*bla*_OXA-58-like_	F	AAGTATTGGGGCTTGTGCTG	52°C	599	Nie (2013)
	R	CCCCTCTGCGCTCTACATAC			
KPC qPCR	F	AACCTCGTCGCGGAACCAT	55°C	141	This study
	R	GCCCTTGAATGAGCTGCACA			
TEM qPCR	F	TTCCGGCTGGCTGGTTTATT	55°C	127	This study
	R	TGACTCCCCGTCGTGTAGAT			
SHV qPCR	F	GCCGATGAACGCTTTCCCAT	55°C	52	This study
	R	CGCCGCAGAGCACTACTTTA			
OXA-23-like qPCR	F	TAATGCTCTAAGCCGCGCAA	55°C	81	This study
	R	TTCTCCAATCCGATCAGGGC			
OXA-51-like qPCR	F	TCGGCCTTGAGCACCATAAG	55°C	197	This study
	R	GCCATAACCAACACGCTTCA			

### 2.5. Absolute quantification of β-lactamase expression

A fresh overnight culture of β-lactamase-positive strains was diluted 1:100 in LB broth, which was incubated at 37°C for 3 h, and then adjusted to an optical density (OD) at 600 nm (OD_600_) of 0.2. DNA-free RNAs were extracted with the RNAprotect Bacteria Reagent (QIAGEN, Hilden, Germany) and RNAprep Pure Cell/Bacteria Kit (TIANGEN) according to the manufacturers’ protocols. The complementary DNA (cDNA) of each sample was synthesized from 1 μg of RNA *via* reverse transcription using the PrimeScript RT Master Mix kit (Takara, Beijing, China). The expression of the PCR-positive genes KPC, TEM, SHV, OXA-23-like, and OXA-51-like was investigated using quantitative PCR (qPCR) with TB Green Premix Ex Taq (Takara) in a final volume of 20 μL. Specific primers are shown in Table 1. The qPCR program was run in the Agilent Mx3005P QPCR System (Agilent Technologies, Santa Clara, CA, United States) as follows: initial denaturation for 30 s at 95°C, followed by 40 cycles of 5 s at 95°C, 30 s at 55°C, and 30 s at 72°C. The copy numbers of genes in the positive isolates were enumerated via comparison with a standard curve derived from 10-fold serial dilutions of target DNA templates. The *R*^2^ value of the standard curve was between 0.990 and 0.999. qPCR was performed in triplicate, and the mean was calculated.

### 2.6. Modification of mCIM conditions

To standardize the protocol and enhance the sensitivity of mCIM, different factors, including the volumes of bacteria and broth, as well as the incubation time of the 10 μg MEM disk in *A. baumannii* suspension were evaluated. First, instead of being taken by loop, the inoculum was prepared by inoculating fresh overnight bacteria grown on LB agar or in broth into TSB, and further adjusted to an OD_600_ of 8.0 in 1 mL of TSB. Second, the bacteria were centrifuged and resuspended in 200 μL TSB for incubation of the MEM disk. Finally, the incubation time was extended to 6 h. The subsequent steps were the same as those used in the mCIM. The evaluation criteria for the results were same as those for the mCIM mentioned above.

### 2.7. Statistical analysis

The sample size of isolates was evaluated using the test for paired sensitivities by PASS 11 (NCSS, Kaysville, Utah, United States). The sensitivity, specificity, and accuracy with 95% confidence intervals (CIs) were determined using the online software MedCalc1 (https://www.medcalc.org/calc/diagnostic_test.php; Khuntayaporn et al., 2021). Sensitivity was the probability of testing positive when the disease (drug resistance) was present. Specificity was a probability of testing negative when the disease (drug resistance) was absent. Indeterminate results or invalid results were categorized as false-negative results when they existed in CP isolates and as false-positive results when they existed in CN isolates (Simner et al., 2018). Statistical analysis was performed using GraphPad Prism software version 8.0 (San Diego, CA, United States), and the results were cross-checked by SPSS 22.0 (SPSS Inc., Chicago, IL, United States). The test of normality of carbapenemase expression levels and MIC values was analyzed using Kolmogorov–Smirnov test, and the correlation between them was analyzed using Pearson’s coefficient. The performances of different carbapenemase phenotypic detection methods were analyzed via unpaired *t*-test. Kappa coefficient with 95% CIs and Youden index were calculated by SPSS 22.0 to evaluate the consistency of the five methods relative to the gene detection results. A value of *p* < 0.05 was considered statistically significant for all tests.

## 3. Results

### 3.1. Bacterial information and antimicrobial susceptibility testing

Ninety isolates were identified as *A. baumannii* using MALDI-TOF MS. When detected using the VITEK 2 system, 85.6% (77/90) of isolates were resistant to IPM, among which 76 strains had MICs of >16 μg/mL and one had an MIC of 8 μg/mL. The remaining 13 strains were sensitive to IPM with MICs at 2 μg/mL for three strains and less than 1 μg/mL for 10 isolates. However, 80.0% (72/90) of strains were confirmed to be IPM-resistant using the E-test method, for which the MICs of 52 isolates were ≥ 8 and < 16 μg/mL, and those of 13 isolates and seven isolates were 16 and ≥ 32 μg/mL, respectively. Among the remaining strains, 5.6% (5/90) and 14.4% (13/90) exhibited sensitivity and intermediate sensitivity to IPM, respectively. Only 22.2% (20/90) of strains yielded consistent results in the IPM MICs tested using the two methods (Supplementary Table S1).

Similarly, for MEM, a resistance ratio of 1.1% (1/90) was obtained using the VITEK 2 system, whereas a resistance ratio of 41.1% (37/90) was determined using the E-test. Moreover, the VITEK 2 system identified 6.7% (6/90) of strains as MEM-sensitive and 92.2% (83/90) strains as having intermediate sensitivity. By contrast, 5.6% (5/90) of strains were susceptible and 53.3% (48/90) of strains had intermediate sensitivity to MEM as confirmed using the E-test. The two methods showed 88.9% (80/90) consistency in the results for MEM MICs. Based on the susceptibility to MEM obtained using the E-test, 40 resistant strains and 45 intermediate strains were classified as unsusceptible strains for subsequent experiments. More specific results of the antimicrobial susceptibility testing are shown in Supplementary Table S1.

### 3.2. Carbapenemase phenotypic detection results of four reported methods

To investigate the mechanism of carbapenem resistance in these clinical strains, meanwhile to confirm the accuracy of these two antimicrobial susceptibility tests, various phenotypic methods for detecting carbapenemases, mainly based on the mCIM, were performed. Among the 90 isolates, only eight were positive for the modified Hodge test and three were intermediate, while 87.8% (79/90) of the strains were considered carbapenemase-negative using this method. When investigated using the mCIM, only seven strains were shown to produce carbapenemases, whereas 56.6% (59/90) were negative and 26.7% (24/90) were intermediate. By contrast, the adjusted mCIM method exhibited the highest positive ratio at 53.3% (48/90) in these four methods. Furthermore, 44 strains were identified as carbapenemase-positive using the sCIM, while the remaining strains were intermediate. The specific results for all the strains tested using four methods are shown in Supplementary Table S1.

### 3.3. PCR detection of β-lactamase-encoding genes

Because the positive ratio of phenotype detection methods was too low to be in accordance with the susceptibility testing results, the existence of 14 β-lactamases genes was investigated using PCR. Five genes, namely, *bla*_TEM_, *bla*_SHV_, *bla*_KPC_, *bla*_OXA-23-like_, and *bla*_OXA-51-like_, were found in 90 isolates at different ratios. Among these genes, *bla*_TEM_ and *bla*_SHV_ encoded penicillinases, while *bla*_KPC_, *bla*_OXA-23-like_, and *bla*_OXA-51-like_ encoded carbapenemases (Bush and Bradford, 2019). The OXA-51-like, TEM, and OXA-23-like genes were detected more frequently than the other two genes, with occurrence ratios of 100.00% (90/90), 87.78% (79/90), and 82.22% (74/90), respectively. Seven out of 90 strains contained the SHV gene, while only one strain contained the KPC gene. The occurrence frequencies of different β-lactamase types are shown in Table 2.

**Table 2:** Occurrence frequencies of different β-lactamase types in 90 clinical *Acinetobacter baumannii* isolates.

β-lactamase numbers	β-lactamase types	Occurrence frequency		Susceptibility to MEM (*n*)
		*n*	%	
One β-lactamase	OXA-51	5	5.56	Sensitive (5)
Two β-lactamases	TEM + OXA-51	6	6.67	Intermediate (6)
	SHV + OXA-51	2	2.22	Intermediate (2)
	OXA-23 + OXA-51	4	4.44	Intermediate (1), Resistant (3)
Three β-lactamases	TEM + SHV + OXA-51	2	2.22	Intermediate (2)
	TEM + OXA-23 + OXA-51	68	75.56	Intermediate (36), Resistant (32)
Four β-lactamases	TEM + SHV + KPC + OXA-51	1	1.11	Resistant (1)
	TEM + SHV + OXA-23 + OXA-51	2	2.22	Intermediate (1), Resistant (1)

bla_OXA-51-like_: OXA-51; bla_TEM_: TEM; bla_SHV_: SHV; bla_OXA-23-like_: OXA-23; bla_KPC_: KPC; and meropenem: MEM.

According to Liu et al. (2018), CP strains were defined as strains that were resistant to carbapenem and harbored carbapenemase genes, while the CN strains were defined as strains that were sensitive to carbapenem or resistant to carbapenem due to other mechanisms. Thus, five sensitive isolates were denoted as CN strains and the remaining 85 isolates were denoted as CP strains.

### 3.4. Absolute quantitative detection of β-lactamase expression

The β-lactamase expression levels were evaluated using absolute quantitative real-time PCR to confirm the production of the five positive β-lactamases detected using PCR. The production of β-lactamases was found in all strains, but at different levels. The expression levels of KPC, TEM, SHV, OXA-23-like, and OXA-51-like were found to be 110,615, 1,700–625,617, 741–36,050, 1,663–79,235, and 232–17,962 copies/μL, respectively. The expression levels of carbapenemases in five CN strains were much lower than those in 85 CP strains.

**Figure 1:**
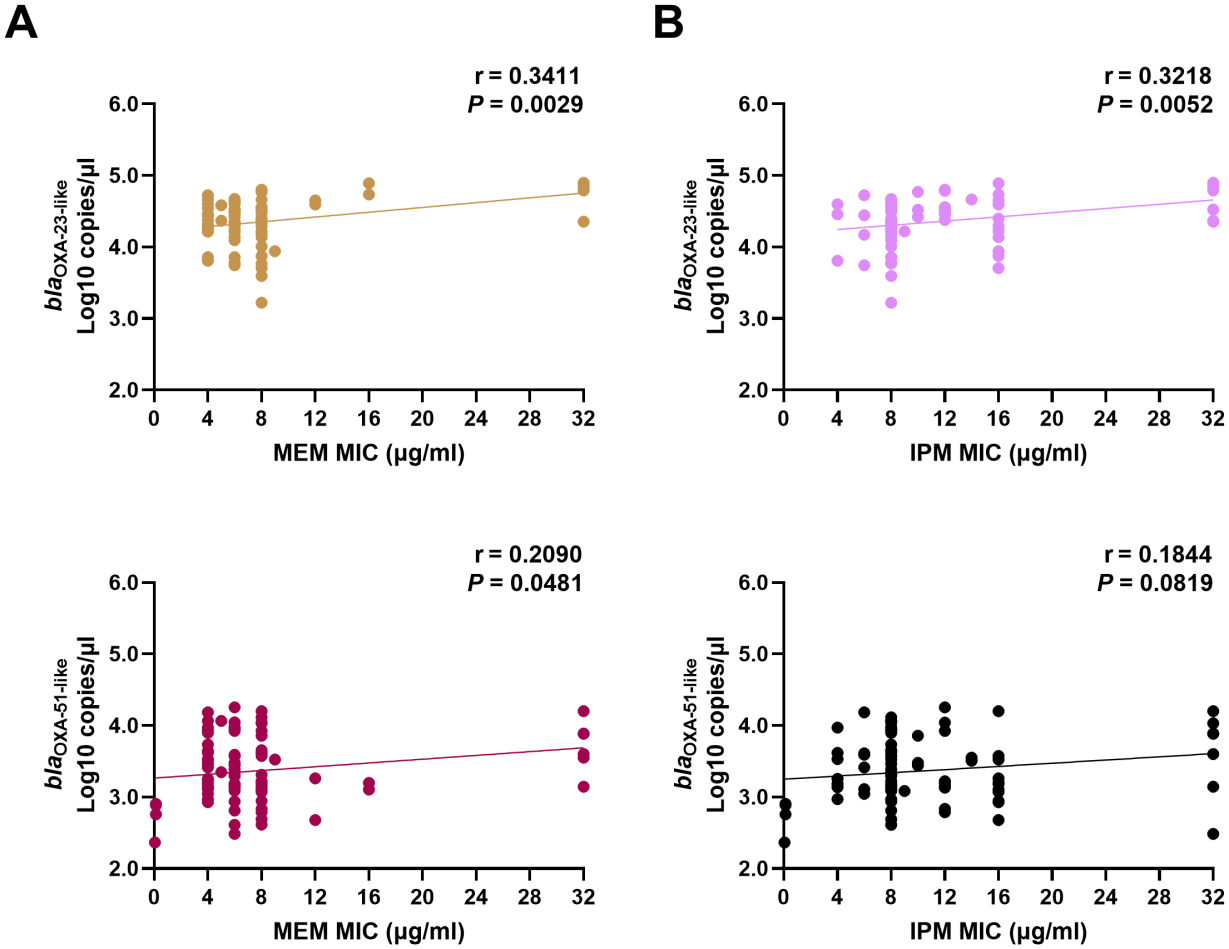
Correlation between carbapenemase expression levels and MIC values of MEM **(A)** and IPM **(B)** in clinical *Acinetobacter baumannii* isolates. The copy numbers of carbapenemase genes (*bla*_OXA-23-like_, and *bla*_OXA-51-like_) from clinical isolates were obtained using real-time PCR. The MICs of MEM and IPM were obtained using the E-test method. The correlation between these two factors was calculated using Pearson’s coefficient. *p* < 0.05 indicates significance. MIC, minimal inhibitory concentrations; MEM, meropenem; IPM, imipenem; and PCR, polymerase chain reaction.

In 85 CP strains, the copy numbers of OXA-23-like were significantly correlated with the MICs of MEM and IPM. The copy numbers of OXA-51-like were correlated with MEM MICs but had no relationship with the IPM MIC values (Figure 1).

### 3.5. Adjustment of oCIM conditions

According to the above gene expression and MIC results, the current phenotypic detection methods including the modified Hodge test, the mCIM, the adjusted mCIM, and the sCIM were not sensitive enough to detect the production of carbapenemases. Therefore, this work aimed to improve the experimental protocols to increase the sensitivity and specificity of the phenotypic method. This study evaluated the influences of a variety of factors, including *A. baumannii* volumes; incubation broth volumes of 200 μL, 400 μL, and 2 mL; and 10 μg MEM disk incubation times of 2, 4, and 6 h (CLSI, 2018; Vu et al., 2020). Ten representative strains with different types of carbapenemases were selected for the pre-experiment to determine the optimal conditions of oCIM. It was anticipated that positive results should follow the standard of the mCIM recommended by the CLSI, where the smaller the diameters of the inhibition zones are, the more likely a positive result is.

**Figure 2:**
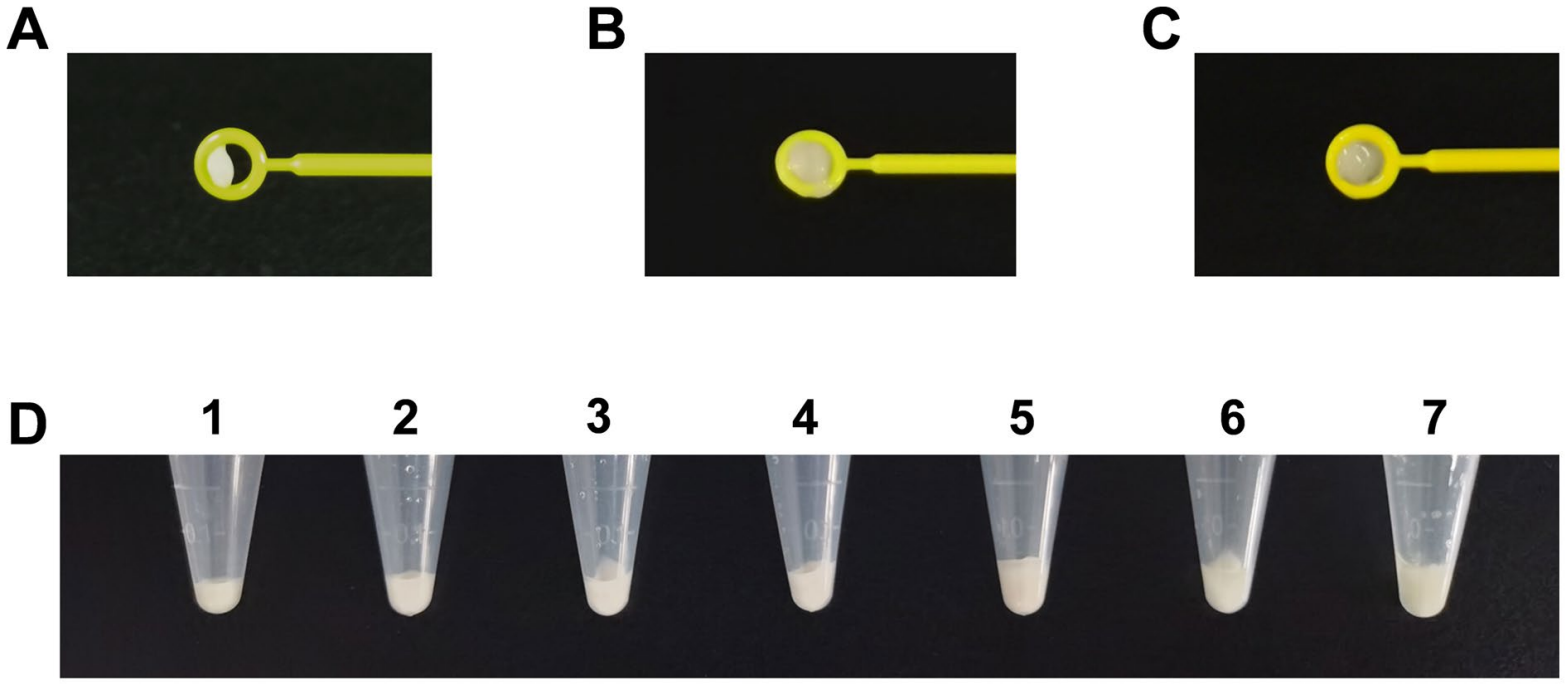
Comparison of different volumes of *Acinetobacter baumannii* collected using different methods for phenotype detection. **(A)** Volumes of bacteria obtained using a 10-μL dry loop. **(B)** Large volumes of bacteria obtained using a 10-μL moist loop. **(C)** Minor volumes of bacteria obtained using a 10-μL moist loop. **(D)** Comparison of different volumes of bacteria obtained using OD_600_ and loops. Tubes 1–5: the bacteria were adjusted to the volume of OD_600_ at 4, 5, 6, 7, and 8. Tubes 6–7: bacteria were collected using one 10-μl moisture loop and two 10-μl moisture loops.

As suggested by the CLSI, tested bacteria were collected using loops. However, there are different collection methods, such as taking bacteria directly from cultural plates (dry loop) or dipping the loop into broth in advance (moist loop). Different volumes of bacteria were obtained using a 10-μL dry loop (Figure 2A) and moist loop (Figures 2B,C). Moreover, even when bacteria were collected using a full 10-μL moisture loop, the volumes could vary depending on different operation methods (Figures 2B,C). By comparison, the same volume of bacteria can be obtained more accurately using OD_600_ as measured by a spectrophotometer. Therefore, different values of OD_600_ were determined at 4, 5, 6, 7, and 8 to compare the bacterial volumes with one and two 10-μL moisture loops as described by Vu et al. (2020). As can be seen in Figure 2D, the bacterial volume of OD_600_ at 6 (No. 3) was close to that of one 10-μL moisture loop (No. 6), and the bacterial volume of OD_600_ at 8 (No. 5) was similar to that of two 10-μL moisture loops (No. 7). Considering clinical practice, the McFarland values of OD_600_ at 6 and 8 were determined. Unexpectedly, it was difficult for the McFarland values to differentiate these two values as both the bacterial density of OD_600_ at 6 and 8 equaled to the McFarland value of 4.00 (Supplementary Figure S1).

**Figure 3:**
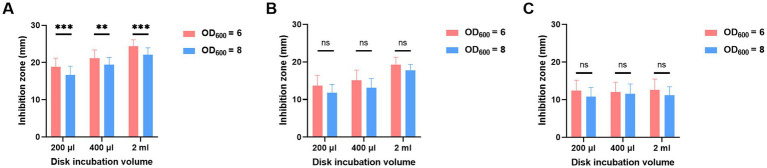
Adjustment of bacterial volumes of oCIM in 10 representative strains. The inoculum of *A. baumannii* adjusted to OD_600_ values of 6 or 8 was suspended in 200 μL, 400 μL, and 2 mL TSB. Then, the 10 μg MEM disks were incubated in bacterial suspensions for 2 h **(A)**, 4 h **(B)**, and 6 h **(C)**, followed by sticking them on the *Escherichia coli* plates. The diameters of the inhibition zones were measured after the overnight incubation of the plates at 37°C. The results are presented as means ± SD. ns, *p* > 0.05; ^**^p < 0.01; ^***^p < 0.001. oCIM, optimized carbapenem inactivation method; TSB, trypticase soy broth; and MEM, meropenem.

To determine which bacteria volume resulted in a smaller inhibition zone, various combinations of incubation volumes and times were tested for OD_600_ of both 6 and 8. As shown in Figure 3, despite MEM disk incubation in 200 μL, 400 μL, and 2 mL of broth and for 2 h (Figure 3A), 4 h (Figure 3B), and 6 h (Figure 3C), the diameters of inhibition zones of OD_600_ at 8 were always smaller than those of OD_600_ at 6.

**Figure 4:**
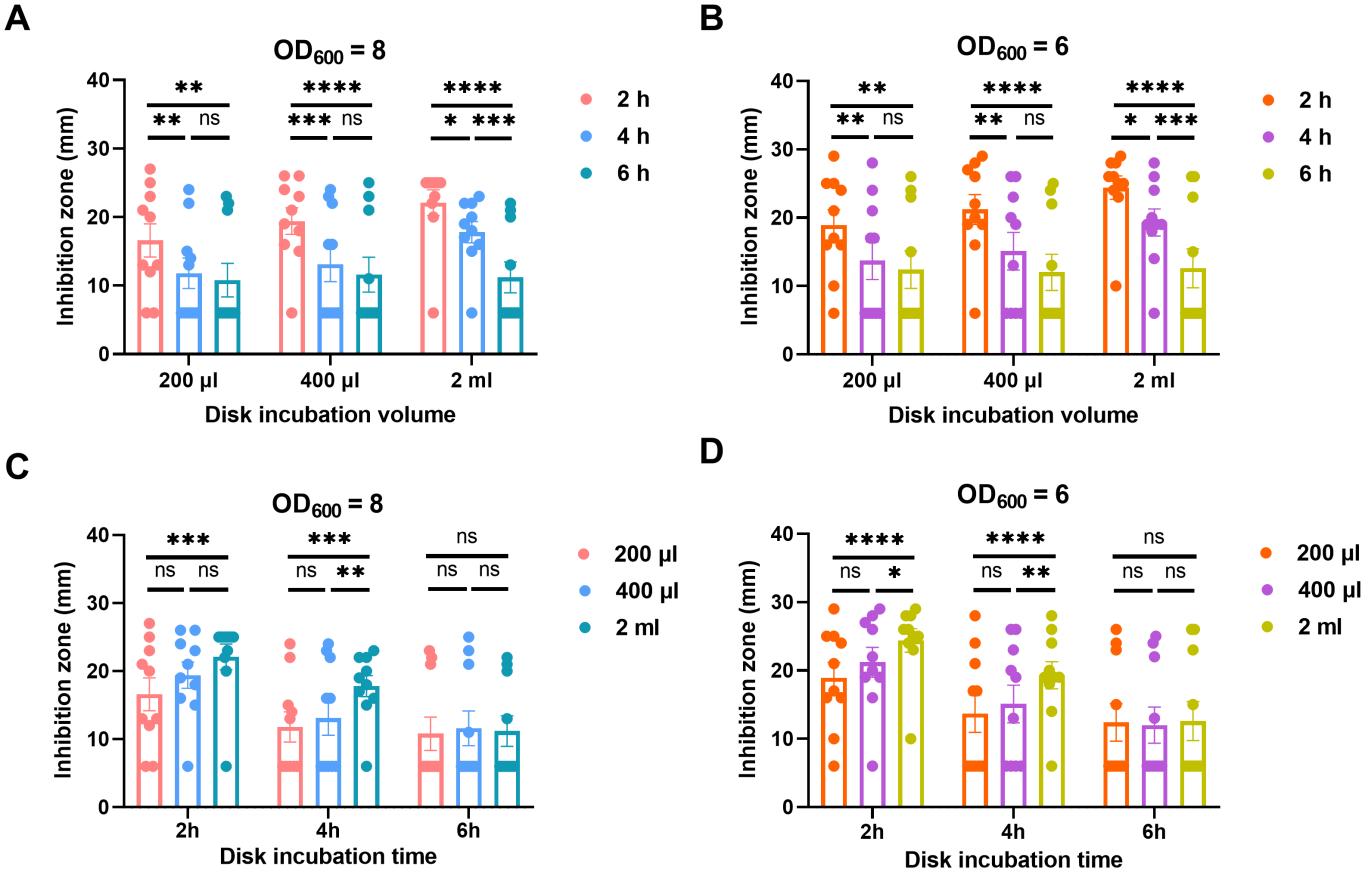
Adjustment of incubation time **(A,B)** and volume **(C,D)** of oCIM in 10 representative strains. **(A,B)**. To compare the performance of different incubation times of 2, 4, and 6 h, the inoculum of *A. baumannii* adjusted to an OD_600_ of 8 or 6 was suspended in 200 μL, 400 μL, and 2 mL TSB. Then, the 10 μg MEM disks were incubated in bacterial suspensions for various periods of time before being stuck on the *Escherichia coli* plates. (C and D) To compare the performance of different incubation volumes of 200 μL, 400 μL, and 2 mL, the inoculum of *A. baumannii* adjusted to an OD_600_ of 8 or 6 was suspended in various volumes of TSB. The 10 μg MEM disks were incubated in these volumes for 2, 4, and 6 h before they were stuck on the *E. coli* plates. The diameters of the inhibition zones were measured after the plates were incubated overnight at 37°C. The results are presented as means ± SD. ns, *p* > 0.05; ^*^p < 0.05; ^**^p < 0.01; ^***^p < 0.001, ^****^p < 0.0001. oCIM, optimized carbapenem inactivation method; TSB, trypticase soy broth; MEM, meropenem.

For the incubation time of the MEM disk in the *A. baumannii* suspension, the diameters of inhibition zones obtained after 4 and 6 h of incubation were remarkably smaller than those obtained after 2 h. There was no significant difference of inhibition zones between the incubation times of 4 and 6 h in either 200 or 400 μL broth (Figures 4A,B).

In addition, the incubation volumes of *A. baumannii* suspension at 200 and 400 μL exhibited smaller inhibition zones than those at 2 mL. Although no significant difference was noticed between the results at 200 and 400 μL, the average inhibition zone of the former was smaller than that of the latter. In addition, the statistical difference in the inhibition zone diameters between 200 μL TSB and 2 mL TSB was more significant than that between 400 μL TSB and 2 mL TSB (Figures 4C,D).

Moreover, for the positive control strain *Klebsiella pneumoniae* strain ATCC BAA-1705 and the negative control strain *Klebsiella pneumoniae* ATCC BAA-1706, the inhibition zones were compared with two tested strains (No.1 and No.2) in different conditions, respectively. It is obvious that when the inoculum of *A. baumannii* was adjusted to OD_600_ of 8 and resuspended in 200 μL TSB, in which MEM disks were incubated for 4 and 6 h, the inhibition zones of the three carbapenemase-positive strains are the smallest, and the negative control strains also meets the standard (Supplementary Figure S2).

According to the pre-experiment, the optimal procedure for the oCIM test was as follows: the inoculum of *A. baumannii* was adjusted to an OD_600_ of 8, then was resuspended in 200 μL TSB, in which the 10 μg MEM disk was incubated for 4 h before being stuck on the *E. coli* plates. The results of the pre-experiment for 10 strains are summarized in Supplementary Table S2.

### 3.6. Evaluation of oCIM

Ninety clinical strains were used to validate the oCIM test. Seventy-nine out of 85 CP strains were confirmed to be carbapenemase-positive. Among the remaining six CP strains, one was regarded as carbapenemase-intermediate and another five were determined to be carbapenemase-negative by oCIM. Of these five negative strains, four had MEM MICs of 4 μg/mL, and only one had a MIC of 8 μg/mL. All five CN strains were verified to be CN because they only contained OXA-51-like and the production of carbapenemase in these strains was low. The sensitivity and accuracy of the oCIM test were 92.94 and 93.33%, respectively. These values were much higher than those of the other four carbapenemase detection methods. Furthermore, Kappa value and Youden index showed better performance of oCIM in consistency with β-lactamase gene detection results (Table 3).

**Table 3:** Performance of phenotypic methods for detection of carbapenemase-positive *Acinetobacter baumannii*.

Clinical *A. baumannii* isolates			Carbapenemase-positive isolates	Carbapenemase-negative isolates	Sensitivity	Specificity	Accuracy	Kappa	Youden index
			*n* = 85	*n* = 5	% (95% CI)	% (95% CI)	% (95% CI)	(95% CI)	
Modified Hodge	(*n*)	P	8	0	9.41% (4.15–17.71%)	100.00% (47.82–100.00%)	14.44% (7.92–23.43%)	0.011 (−0.000–0.020)	0.941
		I	3	0					
		N	74	5					
mCIM	(*n*)	P	7	0	8.24% (3.38–16.23%)	40.00% (5.27–85.34%)	10.00% (4.68–18.14%)	−0.064 (−0.142–0.014)	−0.518
		I	21	3					
		N	57	2					
Adjusted mCIM	(*n*)	P	48	0	56.47% (48.02–69.16%)	100.00% (47.82–100.00%)	58.89% (48.02–69.16%)	0.126 (−0.020–0.232)	0.565
		I	26	0					
		N	11	5					
sCIM	(*n*)	P	44	0	51.76% (40.66–62.74%)	0.00% (0.00–52.18%)	48.89% (38.20–59.65%)	−0.110 (−0.202–-0.018)	−0.482
		I	41	5					
		N	0	0					
oCIM	(*n*)	P	79	0	92.94% (85.27–97.37%)	100.00% (47.82–100.00%)	93.33% (86.05–97.51%)	0.594 (0.308–0.880)	0.929
		I	1	0					
		N	5	5					

CI, confidence interval; mCIM, modified carbapenem inactivation method; sCIM, simplified carbapenem inactivation method, oCIM, optimized carbapenem inactivation method; P, positive; I, indeterminate; N, negative; and *n*, number of isolates.

**Figure 5:**
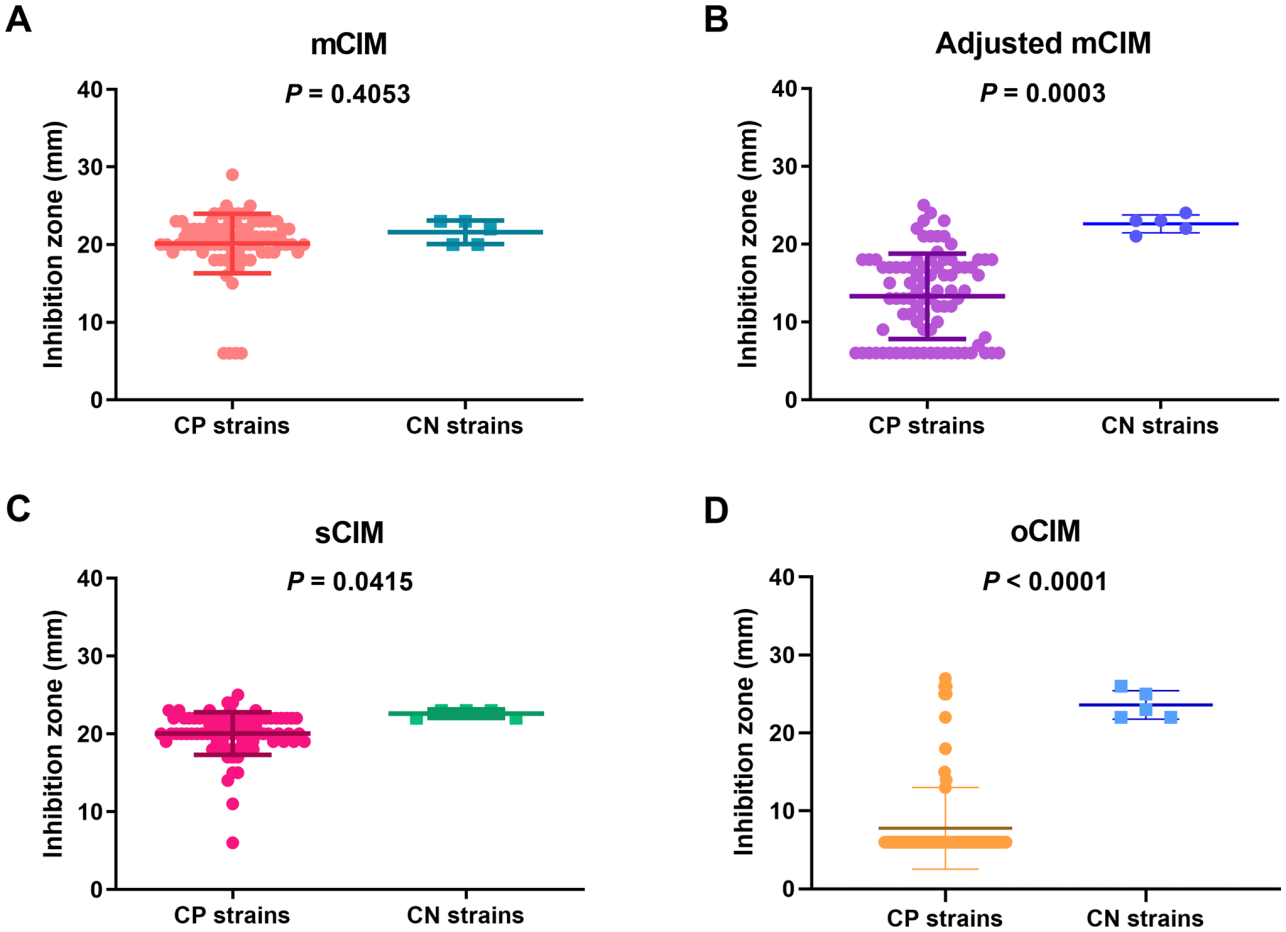
Strain-dependent features of inhibition zones tested using the mCIM **(A)**, adjusted mCIM **(B)**, sCIM **(C)**, and oCIM **(D)** for 85 CP strains and five CN strains. The results are presented as means ± SD. The significance was determined as *p* < 0.05. mCIM, modified carbapenem inactivation method; sCIM, simplified carbapenem inactivation method; oCIM, optimized carbapenem inactivation method; CP, carbapenemase-positive; CN, carbapenemase-negative.

**Figure 6:**
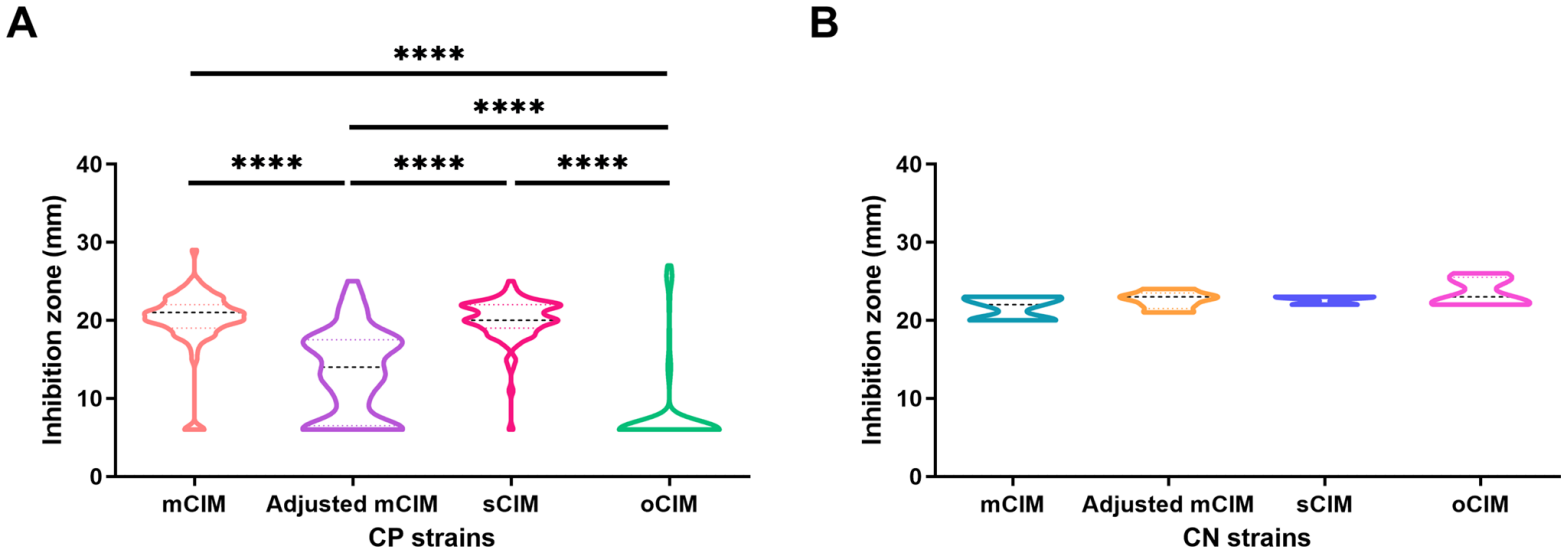
Method-dependent features of inhibition zones among clinical strains. **(A)** Comparison of the diameters of inhibition zones of 85 CP strains obtained by the four methods. **(B)** No difference of the diameters of inhibition zones of five CN strains were observed using the four methods. The results are presented as means ± SD. ^****^p < 0.0001. mCIM, modified carbapenem inactivation method; oCIM, optimized carbapenem inactivation method; sCIM, simplified carbapenem inactivation method; CP, carbapenemase-positive; and CN, carbapenemase-negative.

When evaluating the diameter of the inhibition zones between the CP and CN strains, the methods of the sCIM, the adjusted mCIM, and the oCIM could detect a difference. Among these methods, the oCIM showed higher significance (*p* < 0.0001). However, no significant difference was shown by the mCIM method (Figure 5). In addition, in the CP strains, the average of the diameters tested by oCIM was remarkably smaller than that of the other three methods, with 88.2% (75/85) of strains having diameters of 6 mm (Figure 6A). For the CN strains, the diameters detected using the mCIM, adjusted mCIM, and oCIM were similar and all were above the threshold of negative value, except for in the sCIM (Figure 6B). The negative standard of sCIM was ≥26 mm, based on which all the CN strains were regarded as intermediate. Moreover, the inhibition zones obtained using the oCIM for the positive control strain *K. pneumoniae* strain ATCC BAA-1705 was 6 mm and for the negative control strain *K. pneumoniae* ATCC BAA-1706 was 26 mm, meeting the CLSI evaluation criteria (Supplementary Figure S2A).

### 3.7. Evaluation of performances of different carbapenemase phenotypic detection methods

The overall performances of five carbapenemase phenotypic detection methods are shown in Table 3. The oCIM test was the most sensitive method (sensitivity: 92.94%; 95% CI, 85.27–97.37%), followed by the adjusted mCIM, the sCIM, the modified Hodge test, and the mCIM, among which the mCIM had the lowest sensitivity at only 8.24%. The specificity of the oCIM, the modified Hodge test, and adjusted mCIM (all of which were 100%) was much higher than that of the sCIM (0.00%) and mCIM (specificity: 40.00%; 95% CI, 5.27–85.34%). The accuracy of five detection methods was ranked in descending order as the oCIM (93.33%), adjusted mCIM (58.89%), sCIM (48.89%), the modified Hodge test (14.44%), and the mCIM (10.00%). Nevertheless, the sCIM exhibited the highest intermediate ratio of carbapenemases compared with other four methods (Table 3).

This study further analyzed the performance of carbapenemase detection methods based on carbapenemase types in CP isolates (Table 4). Among all assays, the oCIM test displayed the highest sensitivity in detecting either single or co-existing types of carbapenemases. The *bla_KPC_* gene was present in only one isolate, which was regarded as a CP strain using all five methods. Moreover, the MIC values of IPM and MEM for this strain were 14 and ≥ 32 μg/mL, respectively.

**Table 4:** Performance of phenotypic detection methods according to genotypic characterization for *A. baumannii*.

Clinical	Modified Hodge			mCIM			Adjusted mCIM			sCIM			oCIM			MIC range	
Carbapenemase-positive *A. baumannii* isolates	*n* (%)			*n* (%)			*n* (%)			*n* (%)			*n* (%)			(μg/mL)	
	P	I	N	P	I	N	P	I	N	P	I	N	P	I	N	IPM	MEM
One carbapenemase																	
OXA-51 (*n* = 10)	1 (100.0%)	0	9 (90.0%)	3 (30.0%)	4 (40.0%)	3 (30.0%)	4 (40.0%)	0	6 (60.0%)	5 (50.0%)	5 (50.0%)	0	6 (60.0%)	0	4 (40.0%)	4–12	4
Two carbapenemases																	
KPC & OXA-51 (*n* = 1)	1 (100.0%)	0	0	1 (100.0%)	0	0	1 (100.0%)	0	0	1 (100.0%)	0	0	1 (100.0%)	0	0	14	>32
OXA23 & OXA-51 (*n* = 74)	6 (8.1%)	3 (4.1%)	65 (87.8%)	3 (4.1%)	17 (22.9%)	54 (73.0%)	43 (58.1%)	26 (35.1%)	5 (6.8%)	38 (51.4%)	36 (48.6%)	0	72 (97.2%)	1 (1.4%)	1 (1.4%)	4- > 32	4- > 32

P, positive; I, Indeterminate; N, Negative; and *n*, number of isolates.

## 4. Discussion

For timely and appropriate treatment and proper infection control procedures for *A. baumannii* in clinics, a rapid and accurate phenotypic detection method to determine the carbapenemases in this kind of strain is required. This research group previously investigated the production of carbapenemases in 90 *A. baumannii* clinical strains using currently published phenotypic detection methods, including the modified Hodge test, the mCIM, the adjusted mCIM, and the sCIM. However, low positive ratios were observed in these strains, of which the majority were CRAB. Thus, the MIC values, the existence of carbapenemase-encoding genes, and their expression levels were detected to explain the contradictory results. Moreover, these optimized conditions were determined to construct an improved method, named oCIM, to identify carbapenemase production, and its efficiency was verified using the 90 strains.

The accurate determination of MICs is important because the results can affect the antimicrobial treatment of infectious diseases (Yin et al., 2021). The VITEK2 is the most frequently used automatic system in clinical laboratories to determine the antibiotic MICs. However, its accuracy was not as high as that of E-test strips for both IPM and MEM in the strains tested in the present study. Generally speaking, the IPM MIC obtained using the VITEK2 system was higher than that obtained using E-test strips. Similar results were found for MEM, but normally with lower values obtained using the VITEK2 system than using E-test strips due to the limitation of the tested concentrations in this system. In particular, eight strains were found to be sensitive to IPM using the VITEK2 system, but were found to be resistant using the E-test method. A similar outcome occurred for MEM in one strain. The expression of carbapenemases in these isolates supported the results of the E-test methods. A similar report on colistin resistance in *A. baumannii* was reported, in which it was determined that the performance of VITEK 2 was not appropriate because of the very major error (VME) beyond the acceptable range (Chung et al., 2022). Another study supported these findings and reported that in a comparison with VITEK 2 testing for IPM, E-test strips performed better, and that the VME rates of IPM, MEM, and doripenem obtained by E-test strips were much lower than those obtained using the VITEK 2 system (Markelz et al., 2011). In the present study, both the VITEK 2 system and E-test strips indicated that the resistance rate of IPM was obviously higher than that of MEM. Similarly, in the North-Batinah region of Oman, a recent study showed that the overall resistance rate of IPM was 72% from 2015 to 2019, which was a bit higher than that of MEM at 70% (Sannathimmappa et al., 2021). Due to the influence of other mechanisms, such as a lack of porins or increased efflux pump activity, the MIC does not entirely reflect the production level of carbapenemases (Jacoby et al., 2004).

For carbapenemase-producing *Acinetobacter* spp., it is crucial to quickly inform clinicians of the existence and the type of carbapenemases produced by strains because some agents preferentially exhibit activity against specific carbapenemases (Tamma and Simner, 2018). As in a previous report, PCR screening for carbapenem-resistance genes was taken as the gold standard to evaluate the sensitivity of different phenotypic methods (Abouelfetouh et al., 2019). Indeed, all isolates examined carried the *bla*_OXA-51-like_ gene, which is an intrinsic oxacillinase gene that occurs naturally in *A. baumannii* (Rafei et al., 2015). The *bla*_OXA-23-like_ gene was the second most common carbapenemase gene and was found in 82.22% of isolates, in agreement with previous studies in Jordan (Al-Tamimi et al., 2022) and in China (Ji et al., 2014). Only one strain harbored the *bla*_KPC_ gene, which was supported by another study in which the occurrence of KPC was 0.6% (1/170; Hashemizadeh et al., 2022). However, PCR assay has the disadvantages of high cost and an inability to detect unknown enzymes and is unsuitable for routine laboratory analysis (Doyle et al., 2012). Therefore, an accurate phenotypic detection method is imperative to provide relevant carbapenemase information.

To identify the activity of carbapenemases in the clinical strains in the present study, the most frequently reported four methods were performed. The modified Hodge test did not yield precise positive results; although five types of carbapenemase genes were detected at different expression levels, this method had difficulty distinguishing enzyme activity levels. This might be the reason that, since 2010, the modified Hodge test has only been recommended by the CLSI for detection of KPC-type carbapenemases in *Enterobacterales* (CLSI, 2010), which was in accordance with the result of the present study that the KPC-containing strain was identified as a CP strain. Moreover, some researchers do not recommend the modified Hodge test due to subjective interpretation and performance problems (Kuchibiro et al., 2018; Zhong et al., 2020).

The mCIM is recommended by the CLSI especially for *Enterobacteriaceae* and *Pseudomonas*, and is also useful for *A. baumannii*. As reported by Simner et al., the sensitivity and specificity of mCIM to identify carbapenemase-producing *A. baumannii* were 79.8% (CI, 73–100%) and 52.9% (CI, 89–100%), respectively (Pierce et al., 2017). However, the performance of the mCIM was worse in the present study, which concurred with a recent study published by Howard et al. in which it was found that the sensitivity of the mCIM was as low as 10% (Howard et al., 2020). Another report also confirmed that the mCIM worked poorly for both sensitivity and specificity among CP isolates (Simner et al., 2017). Therefore, the mCIM was unsuitable for the detection of *A. baumannii* carbapenemases (Fan et al., 2020).

The adjusted mCIM and the sCIM were revised based on the mCIM and were reported to exhibit better performance than the mCIM (Jing et al., 2018; Vu et al., 2020). The superiority of the adjusted mCIM compared with the mCIM was verified by the results of the present study, but its sensitivity was much lower than 100%, as reported by Vu et al. (2020). The discrepancy of the results was probably due to the different genotypes of the isolates (Vu et al., 2020). Finally, the sCIM showed a concordance of 51.8% (44/85) with molecular genotypes among the CP strains in the present study, which was approximately half of the value observed by Jing et al. (99.5%; Jing et al., 2018). Negative results were not found in the isolates tested in the present study because the diameters of the inhibition zones ranging from 6 to 25 mm were below the threshold of the negative standard of the sCIM. This conflict has been raised by Jing et al. (2018), who reported that the sCIM may result in false positive results in carbapenem-susceptible isolates due to the weak hydrolysis of IPM by most carbapenemases. Thus, this method would be more suitable for the determination of carbapenem-unsusceptible strains (Jing et al., 2018; Wan et al., 2020).

To date, the activity of carbapenemases in the *A. baumannii* strains tested in the present study could not accurately be determined using the abovementioned newly proposed methods. Therefore, an improved testing method is necessary. The paper-based diffusion method is convenient, low-cost, and widely used in most laboratories (Khuntayaporn et al., 2021). Thus, based on this method, more factors including the number of bacteria, the volume of broth, and the time of incubation were considered to optimize the mCIM experimental conditions.

As mentioned in the CLSI, 1- and 10-μL inoculating loops are recommended to collect *Enterobacteriaceae* and *Pseudomonas aeruginosa*, respectively (CLSI, 2018). According to Simner et al. (2018), increased *A. baumannii* inoculum is required to reliably detect the production of carbapenemases in these non-fermenting bacteria. Indeed, two 10-μL loopfuls of bacteria showed markedly high sensitivity and specificity (Vu et al., 2020). Nevertheless, using a loop to determine the amount of test organisms can be inconsistent even when performed by the same operator and may ultimately lead to inaccurate results (Howard et al., 2020). In addition, the McFarland value could not detect the difference between bacterial numbers in highly concentrated bacterial suspensions. Therefore, the OD_600_ was used to objectively obtain the bacteria. Smaller inhibition zones were obtained with a bacteria volume of OD_600_ at 8, which was similar to that of two 10-μL loops. In addition, decreasing the incubation volume of *A. baumannii* to 200 μL significantly reduced the size of the inhibition zone. This was similar to the suggestion of Lisboa et al. that the concentrated bacteria would accelerate the hydrolysis rate of carbapenemases, thus increasing the mCIM sensitivity (Lisboa et al., 2018; Vu et al., 2020). Furthermore, van der Zwaluw et al. suggested that it was more effective to detect weak carbapenemase activity using an extended incubation time of 4 h instead of 2 h (van der Zwaluw et al., 2015). In the present study, better results were obtained when MEM disks were incubated for 4 and 6 h in *A. baumannii* suspensions. However, 4 h incubation is more appropriate and convenient for clinical practice than incubating for 6 h.

As confirmed based on the clinical isolates tested in the present study, the performance of the oCIM with regard to sensitivity, specificity, and accuracy is superior to that of the other four carbapenemase phenotypic detection methods. Five false-negative results were observed in the present study. This might have been due to the expression levels of some carbapenemase genes, especially *bla*_OXA-23-like_, which exhibited very low expression in one strain and was undetectable in the other four strains. Moreover, these strains were mainly intermediate to MEM. As suggested by Markelz et al. (2011), the *bla*_OXA-23-like_ gene was associated with less sensitivity and increased MICs of carbapenems, which was in accordance with the results of the present study that the MICs of both MEM and IPM were positively correlated with the copy numbers of the *bla*_OXA-23-like_ gene. Therefore, negative strains with intermediate values of MEM might harbor low levels of carbapenemases. In this situation, extending the disk incubation time is recommended to obtain positive results. One special false-negative strain (Strain No. 96) with a very low level of carbapenemases had high MIC values of 8 μg/mL to both MEM and IPM. This may be due to other resistance mechanisms, such as efflux pumps or porins (Potron et al., 2015).

Latest reports also mentioned some newly developed phenotypic methods, such as rCIM-A (Mitteregger et al., 2022) and iCIM (Howard et al., 2020). Different from them, the oCIM was modified and verified based on the expression level of the carbapenemases. In addition, oCIM used the OD_600_ instead of loop to objectively obtain the bacteria, thus to minimize the error caused by human factors.

In conclusion, this study modified and optimized the mCIM test to obtain the improved oCIM. Compared with other carbapenemase phenotypic detection methods, the oCIM is less affected by variables such as laboratory staff and operating experience, which makes it a cost-effective, accurate, and reproducible phenotypic screening method to detect carbapenemase activity. Overall, the oCIM is suitable for detecting carbapenemase-producing *A. baumannii* in clinical microbiology laboratories and can contribute to the control of infections induced by these strains in hospitals.

## Data availability statement

The original contributions presented in the study are included in the article/Supplementary material, further inquiries can be directed to the corresponding authors.

## Author contributions

SZ and PM performed the experiments. SZ and LH drafted the manuscript. JW, PL, KL, SLi, MA-S, JL, SLa, BH, and YC provided assistance in experiments. LH and SH designed the experiment, and provided the overall guidance and revision. All authors contributed to the article and approved the submitted version.

## Funding

This work was funded by the National Natural Science Foundation of China (81702043), the Fundamental Research Funds for the Central Universities (xzy012020050), the “Basic-Clinical” Fusion Innovation Project of Xi’an Jiaotong University (YXJLRH2022020), the Clinical Research Award of the First Affiliated Hospital of Xi’an Jiaotong University (XJT1AF-CRF-2019-015), and the Key Research and Development Program of Shaanxi Province (2017SF-198).

## Conflict of interest

The authors declare that the research was conducted in the absence of any commercial or financial relationships that could be construed as a potential conflict of interest.

## Publisher’s note

All claims expressed in this article are solely those of the authors and do not necessarily represent those of their affiliated organizations, or those of the publisher, the editors and the reviewers. Any product that may be evaluated in this article, or claim that may be made by its manufacturer, is not guaranteed or endorsed by the publisher.

## Supplementary material

SUPPLEMENTARY FIGURE S1 The correlation between the McFarland values and OD_600_ values of bacterial suspensions. The bacterial suspensions of OD_600_ at 6 (A) and that of OD_600_ at 8 (B) were tested using DensiCHEK Plus. Optical density (OD) at 600 nm: OD_600_.

SUPPLEMENTARY FIGURE S2 The inhibition zone diameter results of four strains including 2 tested strains (No.1 and No.2), the negative control strain *Klebsiella pneumoniae* strain ATCC BAA-1706 and the positive control strain *Klebsiella pneumoniae* ATCC BAA-1705 tested in 12 different conditions (*A. baumannii* volumes adjusted in OD_600_ of 8 and 6, incubation volume of *A. baumannii* in 200 μl, 400 μl and 2 mL TSB, and incubation time for 4h and 6h). The labels named 1, 2, 1705 and 1706 on the plates refer to No.1 tested strain, No.2 tested strain, the positive control strain and the negative control strain, respectively.

SUPPLEMENTARY TABLE S1 Information of strains used in this study.

SUPPLEMENTARY TABLE S2 Determination of oCIM conditions.
