# Supplementary figures and images for: The optimized carbapenem inactivation method for objective and accurate detection of carbapenemase-producing Acinetobacter baumannii
Source: Front Microbiol. 2023 Jul 13;14:1185450. doi: 10.3389/fmicb.2023.1185450 (PMC10372451; doi:10.3389/fmicb.2023.1185450)

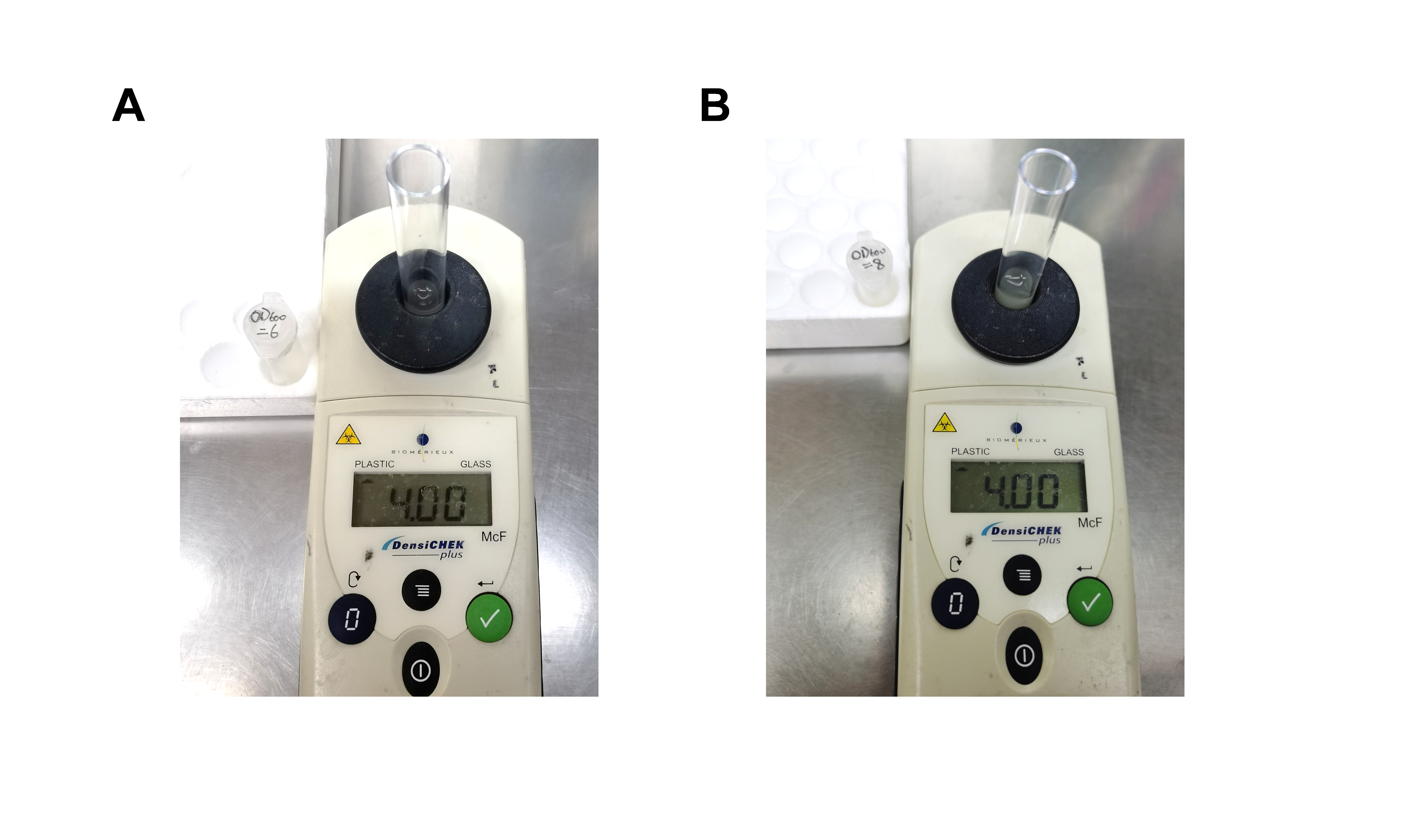

Supplement: SUPPLEMENTARY FIGURE S1 — The correlation between the McFarland values and OD600 values of bacterial suspensions. The bacterial suspensions of OD600 at 6 (A) and that of OD600 at 8 (B) were tested using DensiCHEK Plus. Optical density (OD) at 600 nm: OD600. [file Image_1.TIF]

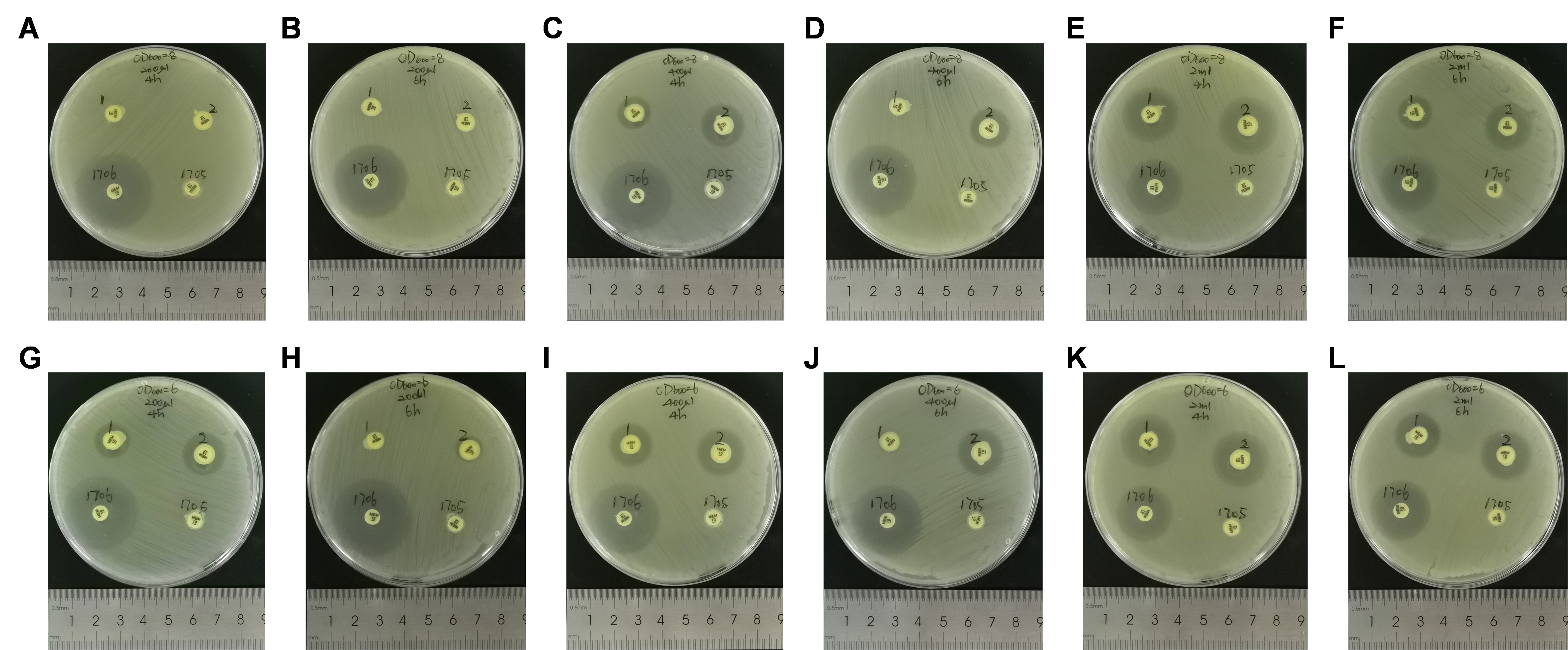

Supplement: SUPPLEMENTARY FIGURE S2 — The inhibition zone diameter results of four strains including 2 tested strains (No.1 and No.2), the negative control strain Klebsiella pneumoniae strain ATCC BAA-1706 and the positive control strain Klebsiella pneumoniae ATCC BAA-1705 tested in 12 different conditions (A. baumannii volumes adjusted in OD600 of 8 and 6, incubation volume of A. baumannii in 200 μl, 400 μl and 2 mL TSB, and incubation time for 4h and 6h). The labels named 1, 2, 1705 and 1706 on the plates refer to No.1 tested strain, No.2 tested strain, the positive control strain and the negative control strain, respectively. [file Image_2.TIF]
